# The Effectiveness of Mechanical Thrombectomy Following the Siriraj Stroke Fast Track Activation Protocol

**DOI:** 10.3390/diagnostics16213654

**Published:** 2026-11-05

**Authors:** Niimron Nisahoh, Boonrerk Sangpetngam, Ekawut Chankaew, Thaweesak Aurboonyawat, Anchalee Churojana, Pensiri Srishewachart, Sakda Sathirareuangchai, Pattarawit Withayasuk

**Affiliations:** 1 Division of Interventional Radiology, Department of Radiology, Faculty of Medicine, Siriraj Hospital, Mahidol University, Bangkok 10700, Thailand; pminor158@gmail.com (N.N.); dboonrerk@gmail.com (B.S.); chankaewek@gmail.com (E.C.); aurboonyawat@gmail.com (T.A.); achurojana@gmail.com (A.C.); 2 Division of Neurosurgery, Department of Surgery, Faculty of Medicine, Siriraj Hospital, Mahidol University, Bangkok 10700, Thailand; 3 Department of Anesthesiology, Faculty of Medicine, Siriraj Hospital, Mahidol University, Bangkok 10700, Thailand; mumu.pensiri@gmail.com; 4 Department of Forensic Medicine, Faculty of Medicine, Siriraj Hospital, Mahidol University, Bangkok 10700, Thailand; sakda.sat@mahidol.edu; 5 Department of Pathology, Faculty of Medicine, Siriraj Hospital, Mahidol University, Bangkok 10700, Thailand

**Keywords:** acute ischemic stroke, large vessel occlusion, mechanical thrombectomy, stroke outcomes, thrombus composition

## Abstract

**Background/Objectives**: Mechanical thrombectomy (MT) has become the standard treatment for ischemic stroke patients with large vessel occlusion (LVO). However, there is limited understanding of the factors influencing outcomes, particularly in the Thai population. This study aimed to evaluate the effectiveness of MT following the Siriraj Stroke Fast Track Activation Protocol and to identify predictors of favorable outcomes. **Methods**: A single-center prospective cohort study enrolled 120 consecutive patients with LVO who underwent MT between April 2023 and August 2024. The primary outcome was favorable functional status (modified Rankin Scale 0–2) at 3 months. Univariate and multivariate logistic regression analyses were performed to identify independent predictors. **Results**: Among 120 patients (median age 68 years; 53% male), 57% achieved favorable outcomes at 3 months. Successful recanalization (modified Treatment in Cerebral Ischemia ≥ 2b) was achieved in 85%, with first-pass effect in 42%. The mortality rate was 7.5%, and symptomatic intracranial hemorrhage occurred in 3.3%. Multivariable analysis was restricted to pre- and intra-procedural variables and estimated by Firth’s penalized-likelihood method. Independent predictors of favorable outcome were successful recanalization (aOR 19.51, 95% CI 2.47–274.05; *p* = 0.004), absence of diabetes mellitus (aOR 6.22, 95% CI 1.58–32.87; *p* = 0.008), lower baseline NIHSS (aOR 0.87 per point, 95% CI 0.76–0.96; *p* = 0.005), and red/mixed thrombus composition (aOR 3.06, 95% CI 1.01–9.96; *p* = 0.049). Younger age, higher baseline ASPECTS and a minimum intra-procedural diastolic blood pressure ≥ 55 mmHg showed consistent but non-significant associations. Post-procedural measures such as the 24 h NIHSS were deliberately excluded from the model because they lie on the causal pathway to the 3-month outcome. **Conclusions**: The outcomes of MT at Siriraj Hospital are comparable to those reported in international studies. Successful recanalization, absence of diabetes, lower baseline stroke severity and red/mixed thrombus composition were independently associated with favorable outcome. The thrombus-composition finding is novel in this population but should be regarded as hypothesis-generating and requires confirmation with immunohistochemical and digital-image analysis in larger cohorts. The protocol described here is resource-appropriate and may be transferable to other stroke centers in low- and middle-income settings.

## 1. Introduction

Acute ischemic stroke (AIS) remains a leading cause of morbidity and mortality worldwide. Large vessel occlusion (LVO), a particularly severe form of ischemic stroke, can result in significant neurological deficits. Thrombolytic therapy with recombinant tissue plasminogen activator is recommended for patients who present within 4.5 h of symptom onset [1,2,3,4]; however, its efficacy is limited in cases of LVO [5]. Since 2015, mechanical thrombectomy (MT) has become the standard treatment for patients with LVO [2,3,6,7,8,9]. Several landmark randomized controlled trials, including MR-CLEAN [10], ESCAPE [11], SWIFT PRIME [12], EXTEND-IA [13], and REVASCAT [14], demonstrated the benefit of MT. The HERMES meta-analysis [15] confirmed MT as a highly effective and safe procedure for patients with LVO. In 2018, the DAWN [16] and DEFUSE 3 [17] trials extended the treatment window to 24 h for selected patients with favorable imaging profiles.

In Thailand, the use of MT has grown substantially. Siriraj Hospital, a large tertiary care and stroke center, has developed guidelines and protocols for rapid stroke therapy, with the most recent update in 2020. MT has since become the standard treatment for all ischemic stroke patients with LVO at this institution. Previous retrospective studies conducted at Siriraj Hospital [18,19] reported 46–60% favorable 3-month outcomes and 80–90% successful recanalization rates. Although several factors have been identified as predictors of favorable outcomes [20,21], understanding in the context of the Thai population remains limited. Those earlier institutional analyses were retrospective and relied on data recorded for clinical rather than research purposes; periprocedural physiological detail, anesthetic management and retrieved-thrombus material were therefore either unavailable or incompletely captured, and patients lost to follow-up could not be distinguished from those never scheduled for review. A prospective design with predefined data collection was adopted to address these limitations, to capture variables that are potentially modifiable at the point of care, and to allow retrieved thrombi to be processed systematically rather than opportunistically.

**Figure 1:**
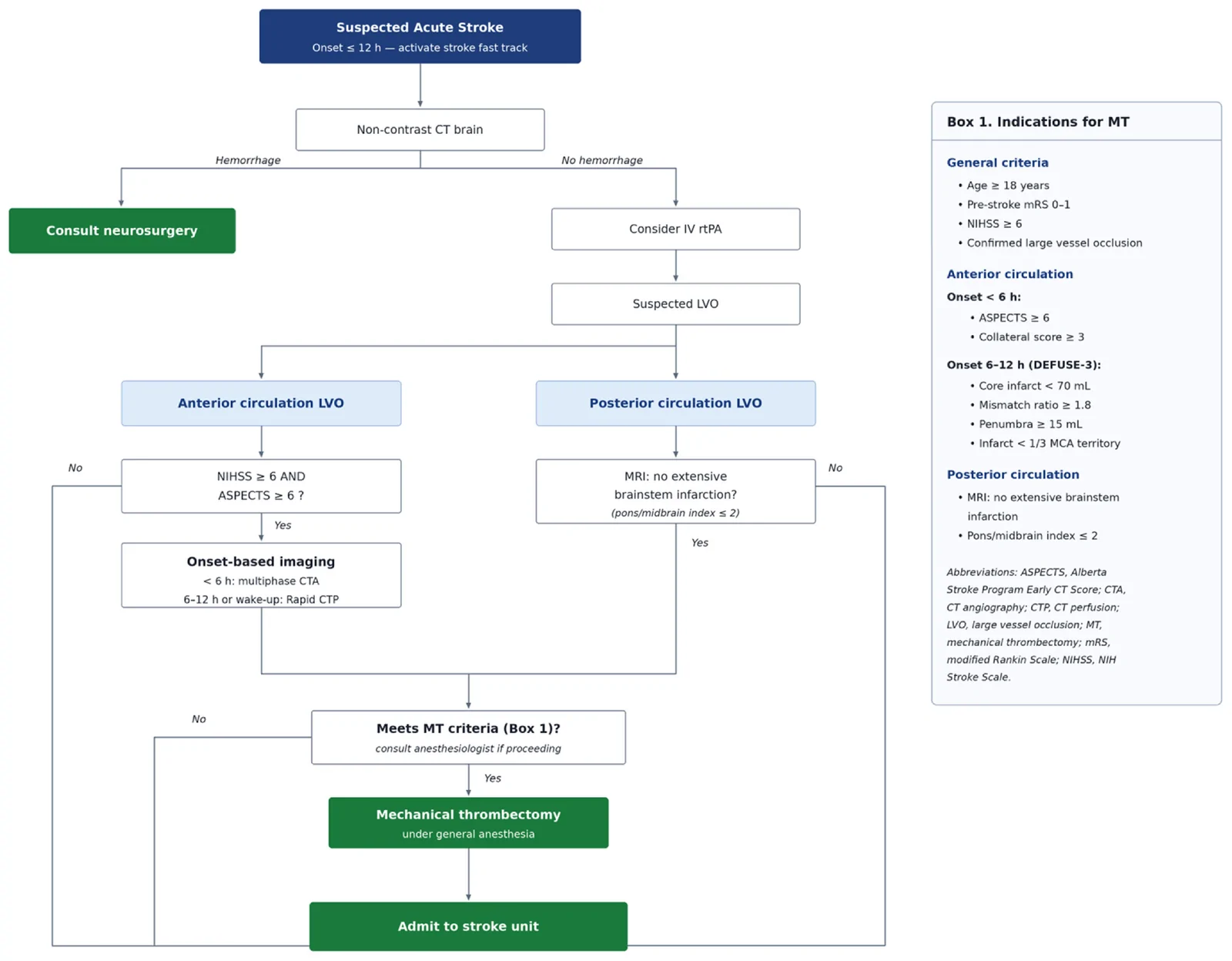
Siriraj Stroke Fast Track Activation Protocol. Arrows indicate the direction of patient flow through the protocol; labels adjacent to arrows leaving a decision box denote the branch taken (“Yes” or “No”). Colour is used to distinguish the role of each node and carries no additional clinical meaning: the dark blue box is the entry point at which the fast track is activated, white boxes are assessment or decision steps, light blue boxes denote the anterior and posterior circulation pathways, and green boxes are terminal actions or endpoints. Box 1 (right) lists the indications for mechanical thrombectomy referenced at the “Meets MT criteria” decision step and is not itself part of the flow.

This study, the first prospective cohort study with comprehensive data collection at our institution, aimed to evaluate the effectiveness of MT and to identify predictors of favorable outcomes following the Siriraj Stroke Fast Track Activation Protocol (Figure 1). The findings provide valuable insights for improving patient selection and optimizing stroke care protocols.

## 2. Materials and Methods

### 2.1. Study Design and Population

This prospective observational cohort study was conducted at Siriraj Hospital from April 2023 to August 2024. We enrolled 120 consecutive adult patients with AIS due to LVO who were candidates for MT following the Siriraj Stroke Fast Track Activation Protocol. The indications for MT are described in Figure 1. For anterior circulation strokes, multiphase computed tomography angiography (CTA) was performed in patients with onset within less than 6 h, and the Alberta Stroke Program Early CT Score (ASPECTS) and collateral scores were assessed. Perfusion imaging was not obtained routinely. Patients presenting within the early window proceeded directly from non-contrast CT and CT angiography to the angiography suite in order to minimize door-to-puncture time; CT perfusion by RAPID software (version 5.2.2; iSchemaView, Inc., Menlo Park, CA, USA) was reserved for patients with late-onset or wake-up stroke and for anterior circulation occlusion (Figure 1), and patient selection in those cases followed the DEFUSE III trial criteria [17]. For patients suspected of having posterior circulation LVO, MT was indicated when magnetic resonance imaging (MRI) of the brain (if available) demonstrated no extensive brainstem infarction. This study was conducted and reported in accordance with the Strengthening the Reporting of Observational Studies in Epidemiology (STROBE) guidelines. Inclusion criteria were: age ≥ 18 years; acute ischemic stroke with angiographically confirmed large vessel occlusion involving the internal carotid artery, M1 or M2 segment of the middle cerebral artery, basilar artery, or vertebral artery; pre-stroke modified Rankin Scale (mRS) ≤ 2; and presentation within the institutional treatment window (≤6 h for standard anterior circulation cases; up to 12 h with favorable perfusion mismatch per DEFUSE 3 criteria; and posterior circulation cases with clinical–radiological mismatch and absence of extensive brainstem infarction on MRI). Exclusion criteria were pre-stroke mRS ≥ 3, extensive established infarction on baseline imaging, contraindications to mechanical thrombectomy or general anesthesia, and refusal of consent. No lower limit of baseline NIHSS was applied. Five patients (4.2%) presented with a baseline NIHSS below 6; four of these had posterior circulation occlusion (vertebral artery in two, basilar artery in two), in which the NIHSS weights cortical function heavily and is recognized to underestimate both the severity of the deficit and the risk of subsequent deterioration, and the remaining patient had an angiographically confirmed M1 occlusion with a disabling hemiparesis. All five were treated on the basis of angiographically confirmed large vessel occlusion together with a disabling or potentially deteriorating deficit rather than on the NIHSS score alone. The institutional review board approved the study, and informed consent was obtained from all patients or their legal representatives.

### 2.2. Data Collection

Comprehensive data were collected, including the following categories:

#### 2.2.1. Baseline Variables

These included demographic information (age, sex, body mass index), medical history (comorbidities), pre-stroke functional status (modified Rankin Scale [mRS]), clinical presentations (including initial National Institutes of Health Stroke Scale [NIHSS] score and Glasgow Coma Scale [GCS] score), laboratory parameters (complete blood count, renal function, blood glucose, and lipid profile), imaging findings (CT ASPECTS, collateral score, hyperdense clot sign, leukoaraiosis, and CT perfusion profile), procedural details, and clinical outcomes. All imaging assessments—including ASPECTS, collateral score, hyperdense clot sign, and leukoaraiosis grading—were independently reviewed and graded by a single neurointerventional radiologist with more than 15 years of experience in cerebrovascular imaging; inter-rater reliability was therefore not assessed.

#### 2.2.2. Procedural Details

**Figure 2:**
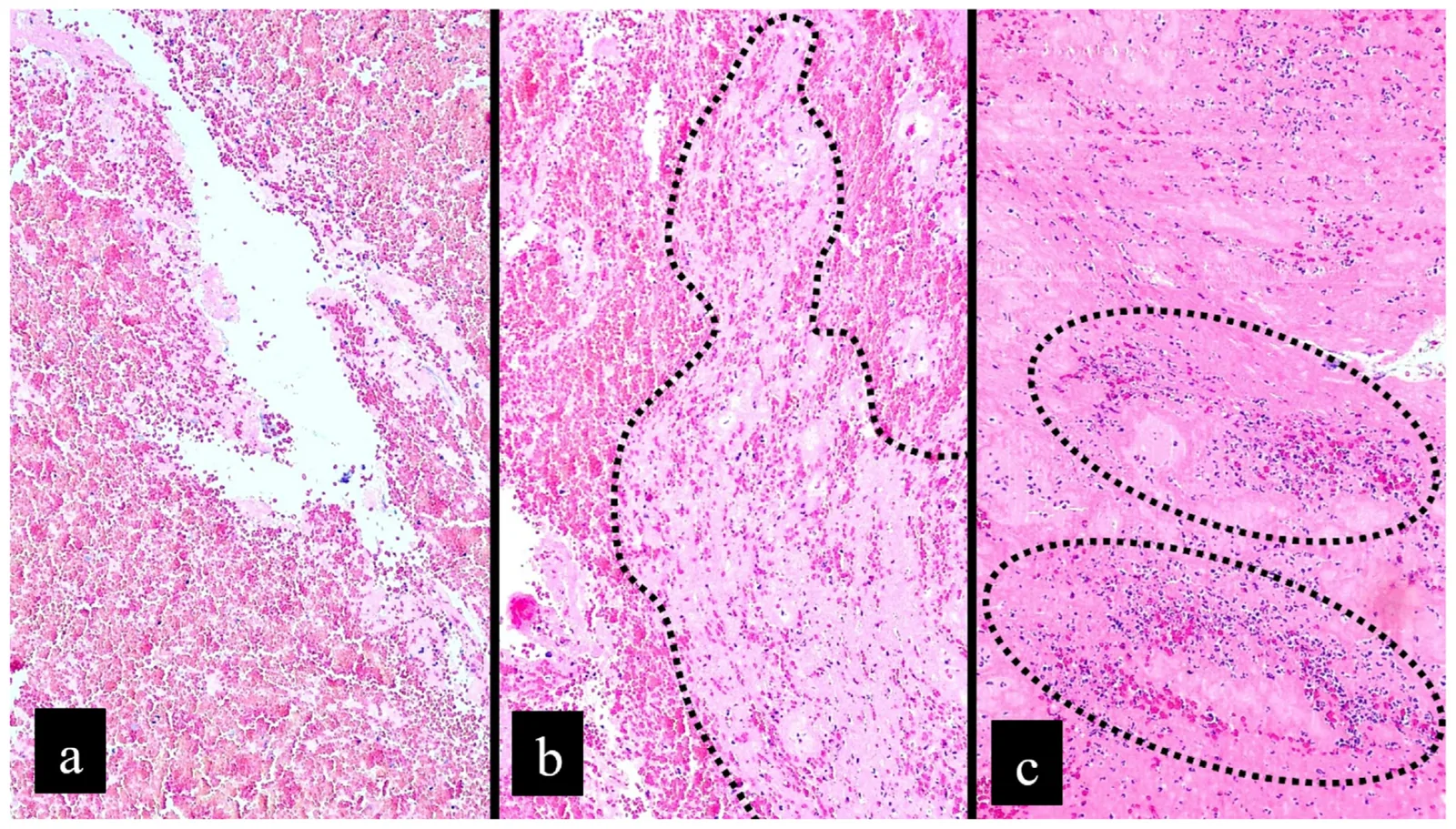
Thrombus composition. Representative microscopic photographs demonstrate thrombus grouping according to composition. (**a**) Red thrombus: RBC-rich thrombus in which the RBC component exceeds the platelet/fibrin component by at least 15%. (**b**) Mixed thrombus: The RBC and platelet/fibrin components are present in approximately equal proportions. The dotted line highlights the platelet/fibrin component. (**c**) White thrombus: Platelet/fibrin-rich thrombus in which the platelet/fibrin component exceeds the RBC component by at least 15%. Rare RBC aggregates are highlighted by dotted ovals. (**a**–**c**) Hematoxylin and eosin stain; original magnification ×100. Sections were stained on a Ventana HE600 automated stainer (Ventana Medical Systems, Inc., Tucson, AZ, USA) and photographed under a Leica DM500 light microscope (Leica Microsystems, Wetzlar, Germany).

All procedures were performed under general anesthesia via transfemoral or, where femoral access was not feasible, alternative arterial access. The first-line thrombectomy technique was selected at the discretion of the treating neurointerventionalist and recorded as direct contact aspiration, the Solumbra technique, stent retrieval, or other techniques including balloon angioplasty. Additional technical details were recorded, including the number of passes, timing parameters, recanalization status, anesthetic management, and complications. Successful recanalization was defined as a modified Treatment in Cerebral Ischemia (mTICI) score [22] ≥ 2b. Retrieved thrombi were sent to the Department of Pathology for hematoxylin and eosin (H&E) staining on an automated slide stainer (Ventana HE600, Ventana Medical Systems, Inc., Tucson, AZ, USA). Histopathological sections were visually examined under routine light microscopy (Leica DM500, Leica Microsystems, Wetzlar, Germany) at multiple magnifications by a single pathologist specialized in thoracic pathology, who classified each specimen into one of three categories based on the relative proportions of cellular components: red (RBC-rich) thrombus, defined as RBC content exceeding fibrin/platelet content by ≥15%; white (fibrin- and platelet-rich) thrombus, defined as fibrin/platelet content exceeding RBC content by ≥15%; or mixed thrombus, defined as RBC and fibrin/platelet contents differing by <15% [23] (Figure 2).

The approximate percentages of RBC and fibrin/platelet content were also estimated. As assessment was performed by a single reader, inter-rater reliability was not evaluated. Postoperative clinical outcomes and complications were recorded. All patients underwent CT brain imaging 24 h post-procedure, and the results were documented.

### 2.3. Outcome Measures

The primary outcome was favorable functional status at 3 months, defined as mRS 0–2. Secondary outcomes were factors influencing favorable functional outcomes. The mRS was assessed in person at the 3-month clinic visit. Six patients (5.0%) did not attend the 3-month visit; for these, the last observed mRS, recorded at 1 month, was carried forward (scores 2, 2, 4, 4, 4 and 5). A complete-case sensitivity analysis restricted to the 114 patients with an observed 3-month mRS is reported alongside the primary analysis.

### 2.4. Statistical Analysis

Descriptive statistics were presented as frequencies, percentages, medians, and ranges. Univariate analysis was performed using Pearson’s chi-squared test, Fisher’s exact test, or the Wilcoxon rank-sum test, as appropriate. Covariates for the multivariable model were specified a priori on clinical grounds rather than by automated stepwise selection, which is known to retain spuriously associated variables and to bias effect estimates away from the null in small samples. Only variables measurable before or during the procedure were eligible: age, sex, diabetes mellitus, baseline NIHSS, baseline ASPECTS, successful recanalization, minimum intra-procedural diastolic blood pressure ≥ 55 mmHg, and thrombus composition. Variables recorded after the procedure—the 24 h and 72 h NIHSS, findings on 24 h CT, and the timing of extubation—were excluded as predictors, because they lie on the causal pathway between recanalization and the 3-month outcome, and conditioning on them attenuates the very treatment effect the analysis is intended to estimate; these variables are reported descriptively and in the univariate analysis only. Because the cohort was modest in size and several covariate–outcome cross-tabulations contained sparse or empty cells—most notably baseline NIHSS < 6, in which all five patients achieved a favorable outcome, so that unpenalized maximum likelihood was not identifiable—all multivariable models were estimated using Firth’s penalized-likelihood method. Odds ratios are reported with profile penalized-likelihood 95% confidence intervals and penalized likelihood-ratio *p*-values. Multicollinearity was assessed by variance inflation factors, which were below 1.3 for every covariate in the final models. Prespecified sensitivity analyses comprised a complete-case analysis of the 3-month outcome, a model refitted after excluding patients with baseline NIHSS < 6, and a model additionally adjusted for sex and induction with succinylcholine. Analyses were performed in Python 3.12.3 (Python Software Foundation, Wilmington, DE, USA) using NumPy 2.3.5, SciPy 1.16.3, pandas 2.3.3 and statsmodels 0.14.6. Firth’s penalized-likelihood estimation, including profile penalized-likelihood confidence intervals and penalized likelihood-ratio tests, was implemented following Heinze and Schemper [24]; variance inflation factors were computed with statsmodels. A *p*-value < 0.05 was considered statistically significant; no adjustment for multiple comparisons was applied, and univariate *p*-values are therefore descriptive.

## 3. Results

The key baseline characteristics and procedural outcomes are summarized in Table 1.

### 3.1. Baseline Characteristics

The median age of the 120 patients was 68 years (range 47–92), and 53% were male. Most patients (62%) were aged ≥ 65 years. The most common comorbidities were hypertension (59%), dyslipidemia (43%), atrial fibrillation (36%), and diabetes mellitus (29%). A history of previous stroke was present in 17% of patients. The median baseline NIHSS score was 15 (range 2–31), with 46% having NIHSS ≥ 16. Common clinical presentations included hemisensory loss (88%), hemiparesis (83%), facial palsy (65%), and aphasia (33%). The median onset-to-door time was 153 min (range 5–845). The median blood glucose level was 119 mg/dL (range 53–470), and 10% of patients had initial blood glucose levels exceeding 200 mg/dL.

Thirty-one patients (26%) had symptom onset of more than 6 h or unknown onset. CT perfusion was performed in 21 patients (17.5%), reflecting the protocol-driven, selective use of perfusion imaging described above rather than incomplete assessment: of the patients who underwent perfusion imaging, 15 of the 20 with recorded timing (75.0%) were beyond 6 h from onset, compared with 18 of 98 (18.4%) among those who did not (*p* < 0.001), and the median onset-to-puncture time was 486 min (IQR 364–622) versus 214 min (IQR 150–313; *p* < 0.001). All 21 perfusion studies were performed for anterior circulation occlusion; no patient with posterior circulation occlusion underwent perfusion imaging. The mean volumes of Tmax > 6 s, cerebral blood flow (CBF) < 30%, mismatch volume, and mismatch ratio (RAPID software, version 5.2.2; iSchemaView, Inc., Menlo Park, CA, USA) were 95 mL, 3 mL, 92 mL, and 9.48, respectively. Of these, 16 patients had no core infarction (CBF < 30%).

**Figure 3:**
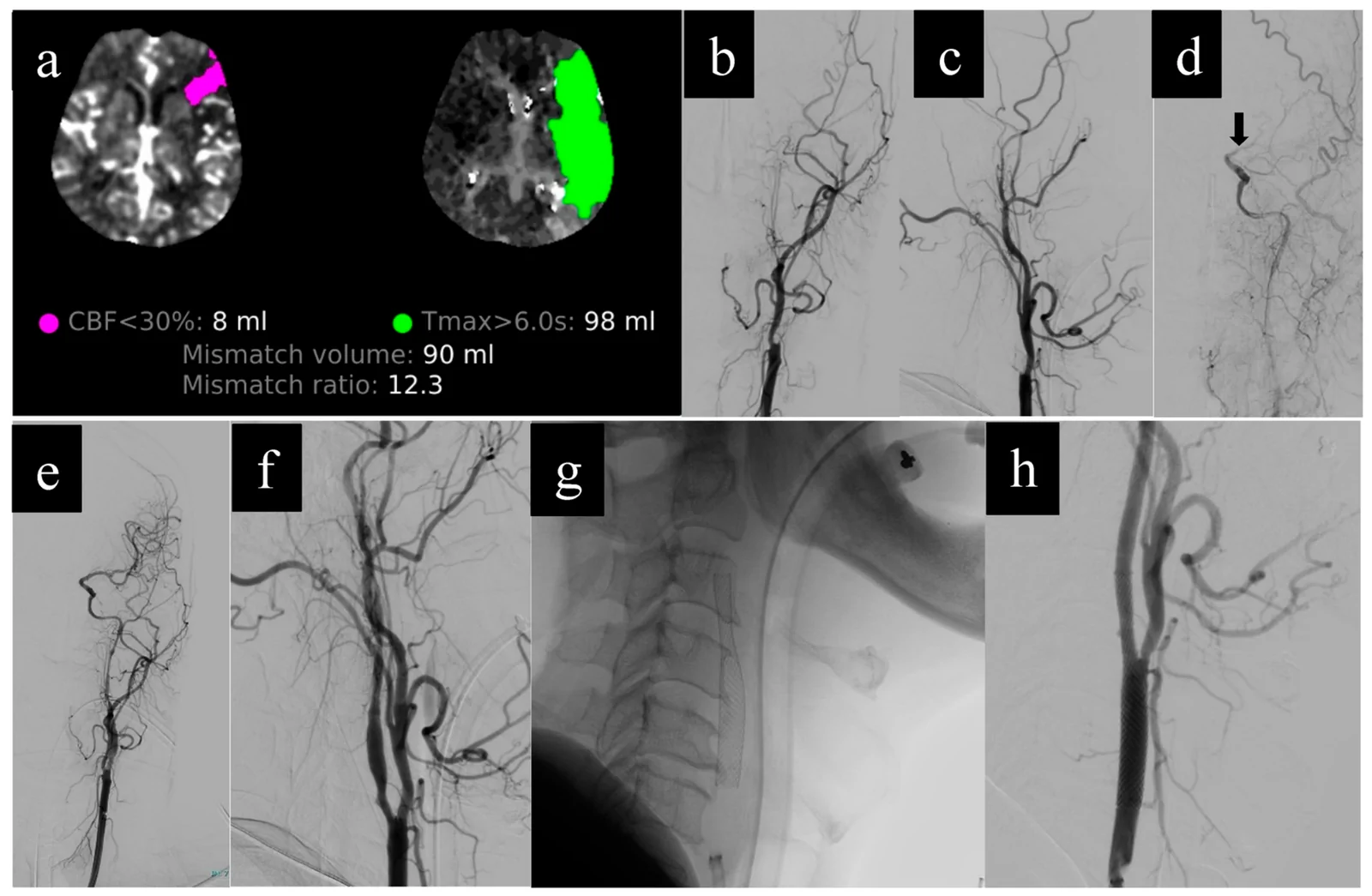
Illustrative case of mechanical thrombectomy for left-sided tandem occlusion with delayed carotid stenting. A 63-year-old man presented with sudden right hemiparesis of unknown time of onset (wake-up stroke); the initial NIHSS score was 14. (**a**) CT perfusion by RAPID software (version 5.2.2; iSchemaView, Inc., Menlo Park, CA, USA) demonstrated a small ischemic core (CBF < 30%) of 8 mL with a mismatch volume of 90 mL and a mismatch ratio of 12.3, consistent with a favorable target mismatch profile. Diagnostic cerebral angiography revealed total occlusion of the left cervical internal carotid artery on (**b**) anteroposterior and (**c**) lateral projections. (**d**) After cervical ICA recanalization with direct contact aspiration and balloon angioplasty, a filling defect was unmasked at the left M1 segment of the middle cerebral artery (arrow), confirming a tandem occlusion. Subsequent direct contact aspiration at the M1 segment achieved complete recanalization (mTICI 3); control angiograms in the (**e**) anteroposterior and (**f**) lateral projections demonstrated full reperfusion with residual stenosis of the cervical ICA. Two weeks later, delayed carotid stenting was performed: (**g**) fluoroscopic working view during stent deployment and (**h**) post-deployment angiogram showing the patent stent and restored ICA flow. At the 3-month follow-up, the patient was functionally independent (mRS 1). Abbreviations: CBF, cerebral blood flow; ICA, internal carotid artery; M1, first segment of the middle cerebral artery; mRS, modified Rankin Scale; mTICI, modified Treatment in Cerebral Ischemia; NIHSS, National Institutes of Health Stroke Scale.

Anterior circulation stroke accounted for 77.5% of cases (*n* = 93, including 9 tandem occlusions; Figure 3), while posterior circulation accounted for 22.5% (*n* = 27). The most common occlusion sites were the middle cerebral artery (MCA; 52%), internal carotid artery (ICA; 18%), and basilar artery (BA; 13%); the remaining sites were the vertebral artery (10%) and tandem occlusions (7.5%) (Table 1). Among anterior circulation strokes (*n* = 93), the median ASPECTS was 7 (range 5–10), with 21 patients (22%) having an ASPECTS of 5–6. Regarding the collateral score, 56% of patients had collateral scores [25] of 4 (range 3–5). A hyperdense clot sign was observed in 66% of cases, predominantly in the M1 MCA segment. Leukoaraiosis [26] was present in 58% of patients, and 28% had calcification of the occluded vessel wall.

### 3.2. Procedural Results

All procedures were performed under general anesthesia. The right common femoral artery was the predominant access site (93%). The first-line techniques used were direct contact aspiration (71%), the Solumbra technique (13%), stent retrieval (10%), and other techniques including balloon angioplasty (5.8%). A rescue technique was required in 51 patients (42.5%) after failure of the first-line approach. Rescue intracranial stenting was performed in 12 patients, corresponding to 23.5% of the 51 patients requiring rescue and 10.0% of the whole cohort; extracranial carotid stenting was performed in a further 6 patients (5.0% of the cohort), so that any rescue stenting was undertaken in 18 patients (15.0%). Rescue stenting was confined to patients with underlying atherosclerotic disease (12 of 34 with intracranial atherosclerotic disease and 6 of 11 with extracranial atherosclerotic disease) and was not required in any patient with a thromboembolic etiology.

The median onset-to-door time was 153 min (range 5–845), the door-to-puncture time was 88 min (range 40–576), the puncture-to-recanalization time was 23 min (range 3–123), and the total procedural time (from patient arrival in the angiography suite to puncture closure) was 75 min (range 25–197).

The most commonly used anesthetic induction agents were propofol (89%) and fentanyl (71%). Succinylcholine was administered in 62% of cases. The typical maintenance agents were sevoflurane (66%) and desflurane (32%). The median blood pressure at baseline, induction, puncture, and post-reperfusion were 151/86, 149/86, 121/71, and 117/66 mmHg, respectively. The maximum and minimum median blood pressures during the procedure were 162/93 and 93/56 mmHg, respectively. Thirty-three percent of patients experienced a mean arterial pressure (MAP) below 70 mmHg for more than 5 min. Patients were extubated immediately in approximately 18% of cases, and an additional 52% were extubated within 24 h.

Overall, successful recanalization (mTICI ≥ 2b) was achieved in 102 patients (85%), with first-pass effect in approximately 42% of cases. Procedure-related complications occurred in 43 patients (36%). The most frequent was distal embolization within the same vascular territory (22.5%, *n* = 27), followed by intracranial vascular tear with subarachnoid hemorrhage (5.0%, *n* = 6), puncture-site complications (4.2%, *n* = 5; 0.8% with and 3.3% without hemodynamic change), distal embolization to a different vascular territory (1.7%, *n* = 2), combined extracranial carotid dissection with intracranial vascular tear (0.8%, *n* = 1), and other procedural events (1.7%, *n* = 2). These categories are mutually exclusive and together account for all 43 patients.

Post-procedure imaging was performed immediately; 79% of patients had no abnormality on cone-beam CT. On 24 h dual-energy CT, 81% of patients showed no evidence of brain herniation, contrast leakage, intraparenchymal hemorrhage, or subarachnoid hemorrhage.

Stroke etiology was classified as thromboembolic (62%), intracranial atherosclerotic disease (ICAD; 28%), extracranial atherosclerotic disease (9.2%), and other or undetermined causes (0.8%). Histopathological analysis was performed on specimens retrieved from 97 patients (81% of the cohort); among these, the distribution was 37% red (RBC-rich), 42% white (platelet-rich), and 21% mixed thrombi. Fresh thrombi (<1 day) were found in 64%, lytic thrombi (1–5 days) in 31%, and organized thrombi (>5 days) in 5.2%.

### 3.3. Clinical Outcomes

Overall, favorable outcomes (mRS 0–2) were achieved in 57% of cases at 3 months (53% for anterior and 67% for posterior circulation strokes). The mortality rate and symptomatic intracranial hemorrhage rate were 7.5% and 3.3%, respectively.

Neurological improvement was observed, with median NIHSS scores decreasing from 15 at baseline to 7 at 24 h, 5 at 72 h, and 3 at discharge. Early neurological improvement (NIHSS < 16) was observed in 82% of patients at 24 h. Additional complications during hospitalization included infection/sepsis (40%), non-symptomatic intracranial hemorrhage (5.8%), and recurrent stroke (5%).

### 3.4. Factors Influencing Favorable Outcomes

Table 2 summarizes factors associated with favorable outcomes in the univariate analysis. Several factors demonstrated significant associations with favorable outcomes, including male sex (62% vs. 42%, *p* = 0.034), younger age (65 vs. 71 years, *p* = 0.009), narrower pulse pressure (62 vs. 73 mmHg, *p* = 0.021), lower initial NIHSS score (13 vs. 17, *p* = 0.005), higher initial GCS score (15 vs. 11, *p* < 0.001), first-pass effect (50% vs. 31%, *p* = 0.041), successful recanalization (94% vs. 73%, *p* = 0.001), shorter puncture-to-recanalization time (21 vs. 30 min, *p* = 0.032), lower immediate postoperative NIHSS score (7 vs. 16, *p* < 0.001), and induction with succinylcholine (72% vs. 48%, *p* = 0.007).

In the prespecified Firth penalized-likelihood multivariable model restricted to pre- and intra-procedural variables and to patients in whom retrieved thrombus was available for histological classification (*n* = 88; 54 favorable, 34 unfavorable), four variables were independently associated with a favorable outcome (Table 3): successful recanalization (adjusted OR [aOR] 19.51, 95% CI 2.47–274.05; *p* = 0.004), absence of diabetes mellitus (aOR 6.22, 95% CI 1.58–32.87; *p* = 0.008), lower baseline NIHSS (aOR 0.87 per 1-point increase, 95% CI 0.76–0.96; *p* = 0.005), and red/mixed thrombus composition (aOR 3.06, 95% CI 1.01–9.96; *p* = 0.049). Younger age (aOR 0.95 per year, 95% CI 0.89–1.00; *p* = 0.054), a minimum intra-procedural diastolic blood pressure ≥ 55 mmHg (aOR 3.28, 95% CI 0.96–13.07; *p* = 0.058) and higher baseline ASPECTS (aOR 1.29 per point, 95% CI 0.90–1.88; *p* = 0.166) showed associations in the expected direction that did not reach statistical significance. All variance inflation factors were below 1.3. In the corresponding model fitted to the whole cohort without thrombus composition (*n* = 107), the same pattern was observed, with narrower intervals: successful recanalization aOR 7.28 (95% CI 1.85–36.31; *p* = 0.004), absence of diabetes aOR 6.22 (95% CI 2.12–21.45; *p* < 0.001), baseline NIHSS aOR 0.89 per point (95% CI 0.81–0.96; *p* = 0.002), and age aOR 0.95 per year (95% CI 0.90–0.99; *p* = 0.023). Results were stable across the prespecified sensitivity analyses. Excluding the five patients with baseline NIHSS < 6 changed no estimate materially (successful recanalization aOR 6.85, 95% CI 1.75–34.04). In the complete-case analysis of the 114 patients with an observed 3-month mRS, successful recanalization (aOR 18.30, 95% CI 2.33–278.41; *p* = 0.004), absence of diabetes (*p* = 0.019) and red/mixed thrombus (aOR 3.59, 95% CI 1.12–12.77; *p* = 0.032) remained significant, while baseline ASPECTS reached significance (aOR 1.47, 95% CI 1.00–2.26; *p* = 0.050) and baseline NIHSS did not (*p* = 0.071). Adding sex and induction with succinylcholine to the model left the four principal associations unchanged; neither variable was independently associated with outcome (male sex aOR 1.22, 95% CI 0.36–4.08, *p* = 0.748; succinylcholine aOR 2.24, 95% CI 0.68–7.91, *p* = 0.184). Unpenalized maximum-likelihood estimation produced effect estimates in the same direction but of larger magnitude and was not identifiable when the baseline NIHSS < 6 indicator was entered, since all five such patients achieved a favorable outcome and none achieved an unfavorable one—the complete separation that motivated the use of penalization.

## 4. Discussion

**Figure 4:**
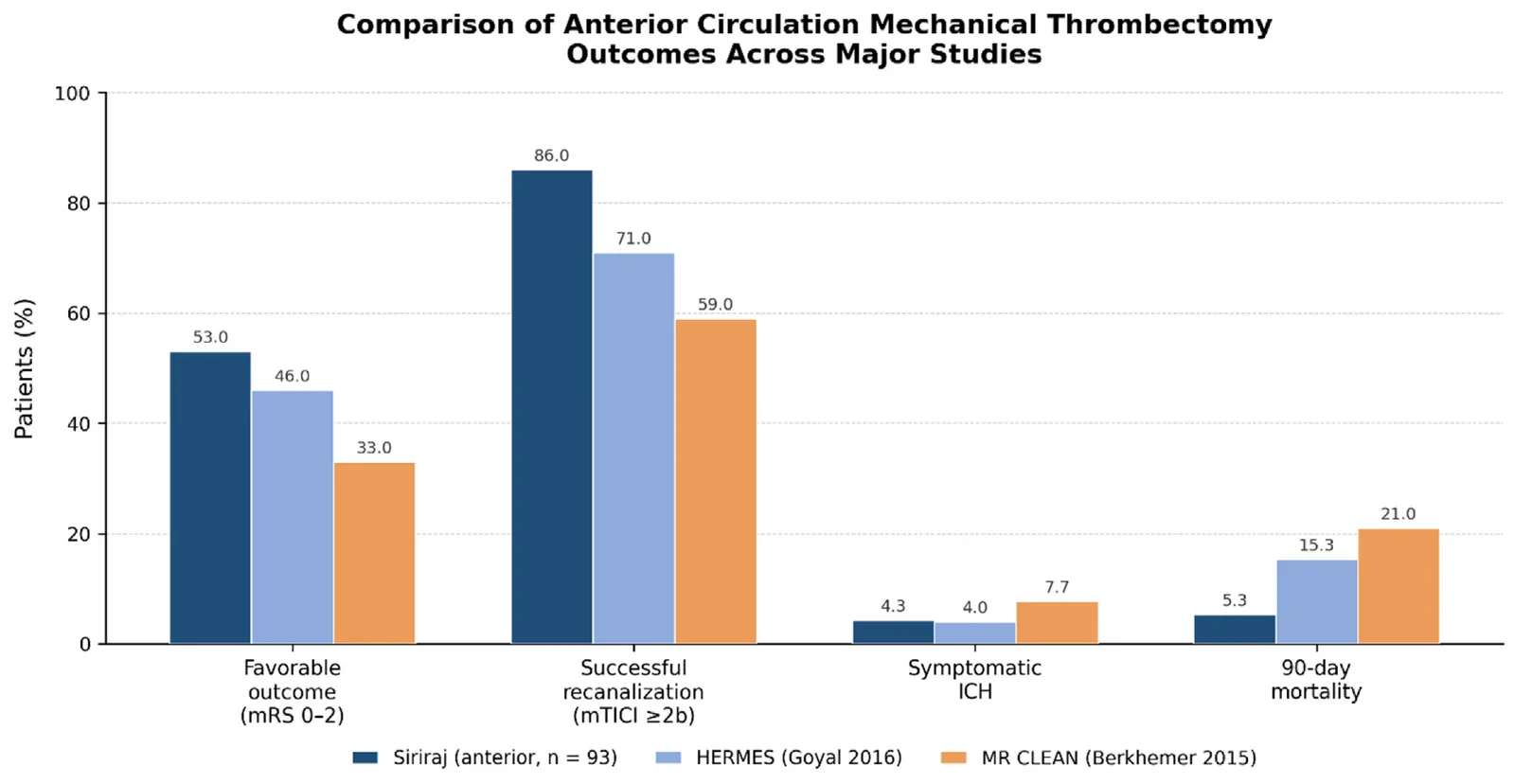
Comparison of anterior circulation mechanical thrombectomy outcomes across major studies: the present cohort, the HERMES meta-analysis [15] and MR CLEAN [10].

This prospective cohort study analyzed outcomes and identified predictors of favorable outcomes following the stroke fast-track protocol at Siriraj Hospital. The overall rate of favorable outcomes was 57%, with 85% successful recanalization. For anterior circulation strokes specifically, favorable outcomes were achieved in 53% of cases, with an 86% successful recanalization rate, which is comparable to previous studies reporting rates between 45–60% [4,15,27,28], as shown in Figure 4.

Regarding the safety profile, our study reported a 7.5% mortality rate and a 3.3% symptomatic intracranial hemorrhage rate, which are lower than those reported in previous studies [15]. The HERMES meta-analysis [15] reported 46% favorable outcomes, 71% successful recanalization, a 15.3% mortality rate, and 4.4% symptomatic intracranial hemorrhage. These findings highlight the efficacy of MT at our institution.

For posterior circulation strokes, our study reported better outcomes with MT (67% vs. 53% for anterior circulation). This finding may be attributable to the lower initial NIHSS scores in patients with posterior circulation strokes (median NIHSS 10 vs. 15 in anterior circulation), which is consistent with previous reports showing that a lower baseline NIHSS score is an independent predictor of favorable outcome after posterior circulation thrombectomy [29]. However, the indications for and effectiveness of MT in posterior circulation strokes remain debatable and warrant further investigation.

Our multivariate analysis revealed four key independent predictors of favorable outcomes, which are consistent with the existing literature while also providing novel insights.

Regarding baseline characteristics, the absence of diabetes mellitus was the strongest comorbid predictor of favorable outcome (aOR 6.22), consistent with previous reports [21,30]. Male sex was associated with favorable outcome in the univariate analysis (62% vs. 42%, *p* = 0.034) but was not independently associated once pre- and intra-procedural covariates were accounted for (aOR 1.22, 95% CI 0.36–4.08; *p* = 0.748). The literature is divided on this point, with some studies reporting a male advantage and others finding no difference [27,31]. Our data are more consistent with the latter, and we would caution against interpreting the univariate association as an independent effect.

Lu et al. [30] reported lower odds of functional independence (aOR 0.48) in patients with diabetes mellitus. Diabetes is associated with more severe small vessel disease and poorer collateral circulation, which contributes to worse outcomes. Our findings also demonstrated that an initial blood glucose level below 200 mg/dL was associated with better outcomes in the univariate analysis, underscoring the importance of glycemic control.

Successful recanalization (mTICI ≥ 2b) was the strongest independent predictor of favorable outcome (aOR 19.51 in the pathology subgroup and 7.28 in the whole cohort), consistent with several studies [15,20,31]. The HERMES meta-analysis [15] reported that successful recanalization was associated with improved outcomes. In the univariate analysis, the first-pass effect was also associated with better outcomes (50% vs. 31%, *p* = 0.041), as was a shorter puncture-to-recanalization time; neither retained an independent association once successful recanalization was included in the model, indicating that their effect on functional outcome operates largely through the achievement of reperfusion rather than independently of it. A meta-analysis by Zaidat et al. [32,33] demonstrated that the first-pass effect is a strong predictor of good outcomes. These findings underscore the importance of technique optimization and device selection as means of achieving reperfusion.

Two aspects of anesthetic management deserve comment. A minimum intra-procedural diastolic blood pressure ≥ 55 mmHg was associated with favorable outcome in the univariate analysis (72% vs. 54%, *p* = 0.054; median minimum DBP 57 vs. 55 mmHg, *p* = 0.018) and retained a substantial but not statistically significant association in the adjusted model (aOR 3.28, 95% CI 0.96–13.07; *p* = 0.058). We report this as a signal rather than as an established predictor: the direction is consistent with the physiological expectation that maintaining adequate cerebral perfusion pressure protects the penumbra, but the present cohort is not large enough to establish it. Early extubation within 24 h showed a strong univariate association (88% vs. 44%, *p* < 0.001) but was not entered into the multivariable model, since patients who remain intubated do so largely because they are already doing badly; this variable is best understood as a marker of early clinical trajectory rather than a modifiable determinant of outcome. Petersen et al. [34] reported that 87% of patients experienced MAP reductions during MT, which were associated with larger infarct volumes and poor outcomes. All patients in our study underwent MT under general anesthesia. Previous randomized controlled trials have demonstrated that general anesthesia and conscious sedation result in comparable functional outcomes after mechanical thrombectomy [35]. Early extubation has been associated with shorter lengths of stay and better outcomes in various surgical procedures [36,37]. Fandler-Höfler et al. [38] found that shorter ventilation time and earlier extubation were associated with improved outcomes. These findings highlight the importance of meticulous anesthetic care in improving MT outcomes. Additionally, our study found that induction with succinylcholine was associated with better outcomes (72% vs. 48%, *p* = 0.007). A meta-analysis [39] found that succinylcholine is superior to rocuronium for achieving excellent conditions during rapid sequence induction, which may reduce procedure time and improve outcomes.

Early response markers behaved as expected but were deliberately excluded from the multivariable model. A 24-h NIHSS < 16 was present in 67 of 68 patients with a favorable outcome compared with 31 of 52 (60%) of those without, and the absence of significant abnormality on 24 h CT showed a similarly strong association. Both are measured after the intervention and lie on the causal pathway between reperfusion and the 3-month outcome, so treating them as predictors is close to circular: in an exploratory model retaining the 24 h NIHSS, it absorbed the treatment effect entirely, reducing the adjusted odds ratio for successful recanalization to 2.03 (*p* = 0.61) while inflating the estimate for thrombus composition. We therefore present these variables as descriptive measures of early neurological response rather than as predictors, which we regard as the more defensible interpretation. Early neurological improvement reflects successful reperfusion and salvage of the ischemic penumbra. Hendrix et al. [40] identified NIHSS ≤ 8 at 24 h as an independent predictor of favorable outcomes. Our study routinely performed postoperative follow-up imaging, predominantly with dual-energy CT, and found that the absence of hyperdense lesions or brain herniation was an independent predictor of good outcomes. Intracranial hemorrhage after recanalization has been associated with poor outcomes [15,41], particularly symptomatic intracranial hemorrhage. In our cohort, the 3.3% rate of symptomatic intracranial hemorrhage was associated with poor outcomes (4% vs. 0%, *p* = 0.033). Postoperative monitoring of neurological status and imaging should be implemented at all institutions performing MT.

**Figure 5:**
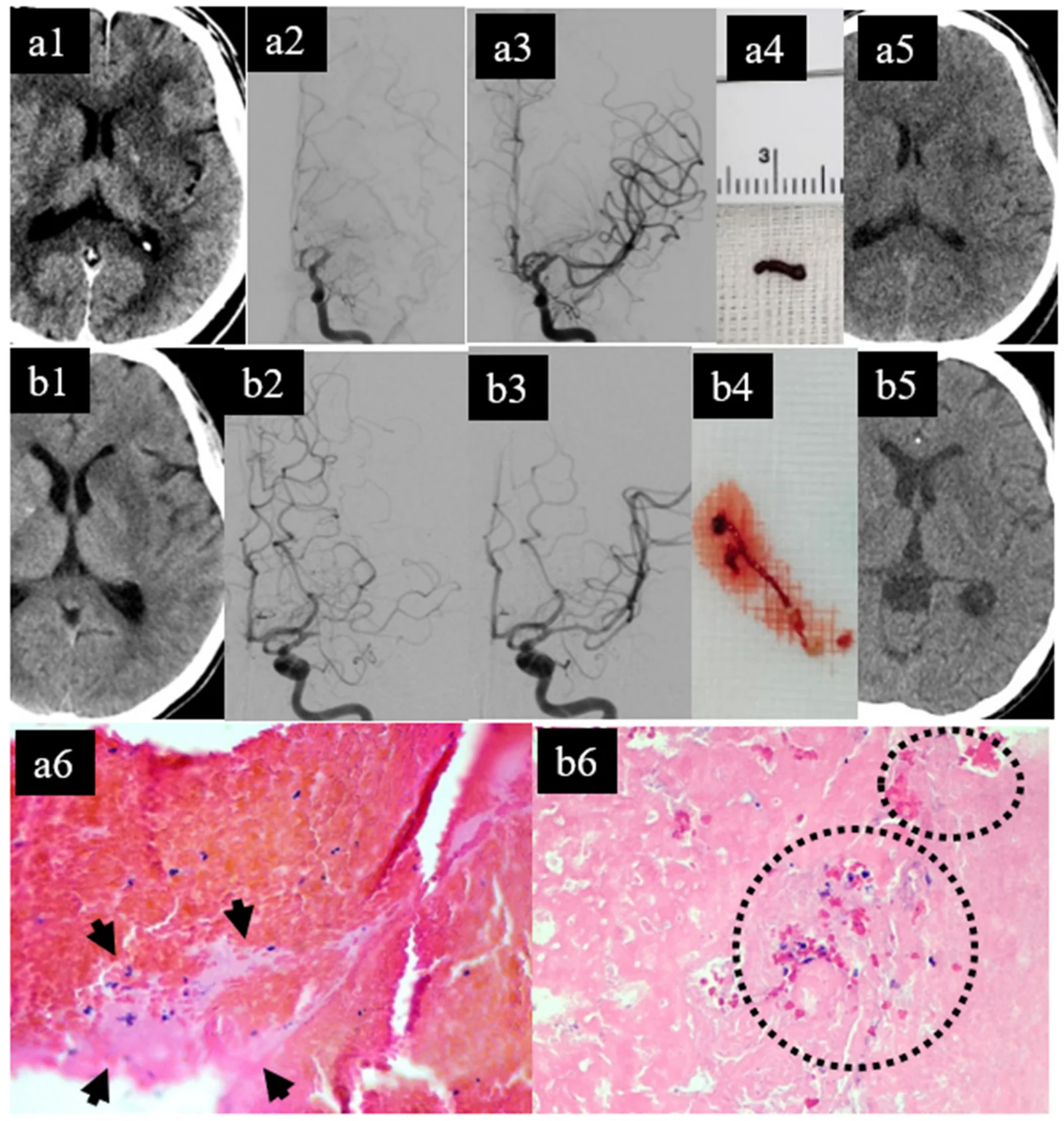
Paired illustrative cases demonstrating the impact of thrombus composition on functional outcome after successful mechanical thrombectomy for M1-segment middle cerebral artery occlusion. Both patients underwent single-pass direct contact aspiration with complete recanalization (mTICI 3) and no infarct progression or hemorrhagic transformation on 24 h follow-up imaging; however, the clinical outcomes differed strikingly. Patient A (top row, (**a1**–**a6**)): a 62-year-old man presented 9 h after symptom onset with right hemiparesis (initial NIHSS 24). (**a1**) Non-contrast CT (NECT) showed an established infarction of the left basal ganglia (ASPECTS = 6). Left internal carotid artery (ICA) angiograms in the anteroposterior projection showed (**a2**) total occlusion of the left M1 segment before treatment and (**a3**) complete recanalization after a single pass of direct contact aspiration. (**a4**) Macroscopic appearance of the retrieved thrombus, predominantly red in color, measuring approximately 5 mm in length; the adjacent ruler is graduated in millimeters. (**a5**) Follow-up NECT at 24 h demonstrated no infarct progression and no hemorrhagic transformation. (**a6**) Histopathological examination of the retrieved specimen demonstrated a red blood cell (RBC)-rich thrombus composed of densely packed, intact erythrocytes with minimal fibrin and platelet components (arrowheads) (hematoxylin and eosin stain; original magnification ×100; Leica DM500, Leica Microsystems, Wetzlar, Germany). The patient improved markedly within 24 h and was functionally independent at 1-month follow-up (mRS 0). Patient B (bottom row, (**b1**–**b6**)): a 48-year-old man presented 1 h after symptom onset with right hemiparesis (initial NIHSS 11). (**b1**) Baseline NECT demonstrated infarction of the left basal ganglia (ASPECTS = 6). Left ICA angiograms showed (**b2**) total occlusion of the left M1 segment and (**b3**) complete recanalization after a single pass of direct contact aspiration. (**b4**) Macroscopic appearance of the retrieved specimen, composed of mixed red thrombus material measuring approximately 6 mm in length together with calcified fragments of approximately 2 mm; dimensions were recorded at gross examination, as no scale reference was included in the photograph. (**b5**) Follow-up NECT at 24 h showed no infarct progression and no hemorrhagic transformation. (**b6**) Histopathological examination of the retrieved specimen demonstrated a fibrin- and platelet-rich (white) thrombus composed predominantly of fibrin and platelets, with sparse erythrocytes (dotted circles) (hematoxylin and eosin stain; original magnification ×100; Leica DM500, Leica Microsystems, Wetzlar, Germany). Despite an early presentation, mild baseline deficit, and identical procedural success, the patient showed no improvement in right-sided weakness and remained severely disabled at 1-month follow-up (mRS 5). These contrasting cases illustrate the independent association between thrombus composition and functional outcome identified in the multivariate analysis (Table 3), supporting the role of histopathological characterization of retrieved emboli as a prognostic biomarker after mechanical thrombectomy. Abbreviations: ASPECTS, Alberta Stroke Program Early CT Score; ICA, internal carotid artery; mRS, modified Rankin Scale; mTICI, modified Treatment in Cerebral Ischemia; NECT, non-contrast computed tomography; NIHSS, National Institutes of Health Stroke Scale; RBC, red blood cell.

A novel finding of this study was the association between red/mixed thrombus composition and better outcomes compared with the white thrombus group (aOR 3.06, 95% CI 1.01–9.96; *p* = 0.049; Figure 5). This association should be regarded as hypothesis-generating rather than established. Specimens were classified by light microscopy of hematoxylin and eosin sections by a single pathologist, without immunohistochemistry, digital image analysis, or a second independent reader, so classification error cannot be excluded and its magnitude cannot be quantified; thrombus was retrievable in 97 of 120 patients (81%), so the estimate derives from a subgroup; and the confidence interval reaches unity. Confirmation in a larger cohort using immunohistochemical characterization and quantitative digital image analysis, read independently by more than one observer, is required before thrombus composition could be considered a prognostic biomarker.

In the univariate analysis, a higher percentage of RBC in the thrombus (median 50% vs. 40%, *p* = 0.015) and a lower fibrin/platelet percentage (median 30% vs. 48%, *p* = 0.002) were associated with better outcomes. RBC-rich thrombi may be more amenable to mechanical retrieval than white thrombi. Previous studies [42,43] have reported that fibrin-rich thrombi are more resistant to retrieval than RBC-rich thrombi, possibly owing to their greater stiffness and adhesion to vessel walls. Further studies examining detailed histopathology using immunohistochemical stains and digital image analysis, along with preoperative imaging parameters, should be pursued.

Several additional factors demonstrated associations in the univariate analysis but did not emerge as independent predictors. Younger age, the absence of leukoaraiosis, shorter procedure times, the absence of postoperative infection/sepsis, and non-ICAD etiology were associated with favorable outcomes. Our cohort found that younger age was associated with better outcomes, particularly among patients aged < 65 years (49% vs. 25% favorable outcomes, *p* = 0.009). This is consistent with previous studies [44,45] reporting that younger patients achieve better functional independence, especially those under 65 years [46], likely due to greater neuroprotective capacity and neuroplasticity [45].

Patients with leukoaraiosis, which may reduce neuroplasticity, had poorer outcomes (44% vs. 74% favorable outcomes, *p* = 0.004). Hancer et al. [47] reported that white matter hyperintensity volume was associated with poorer functional outcomes. Shorter time-to-recanalization and procedure times were also associated with good outcomes. The REVASCAT trial [48] found that a longer time to reperfusion was negatively associated with favorable outcomes, consistent with the “time is brain” principle in acute stroke management.

The absence of postoperative sepsis or infection was significantly associated with better outcomes (74% vs. 42%, *p* < 0.001). This is consistent with studies showing that post-stroke infections are associated with poorer outcomes. The PREDICT study [49] found that preventive antibiotics did not improve functional outcomes but reduced infection incidence. This finding emphasizes the importance of infection prevention strategies in post-procedural care.

**Figure 6:**
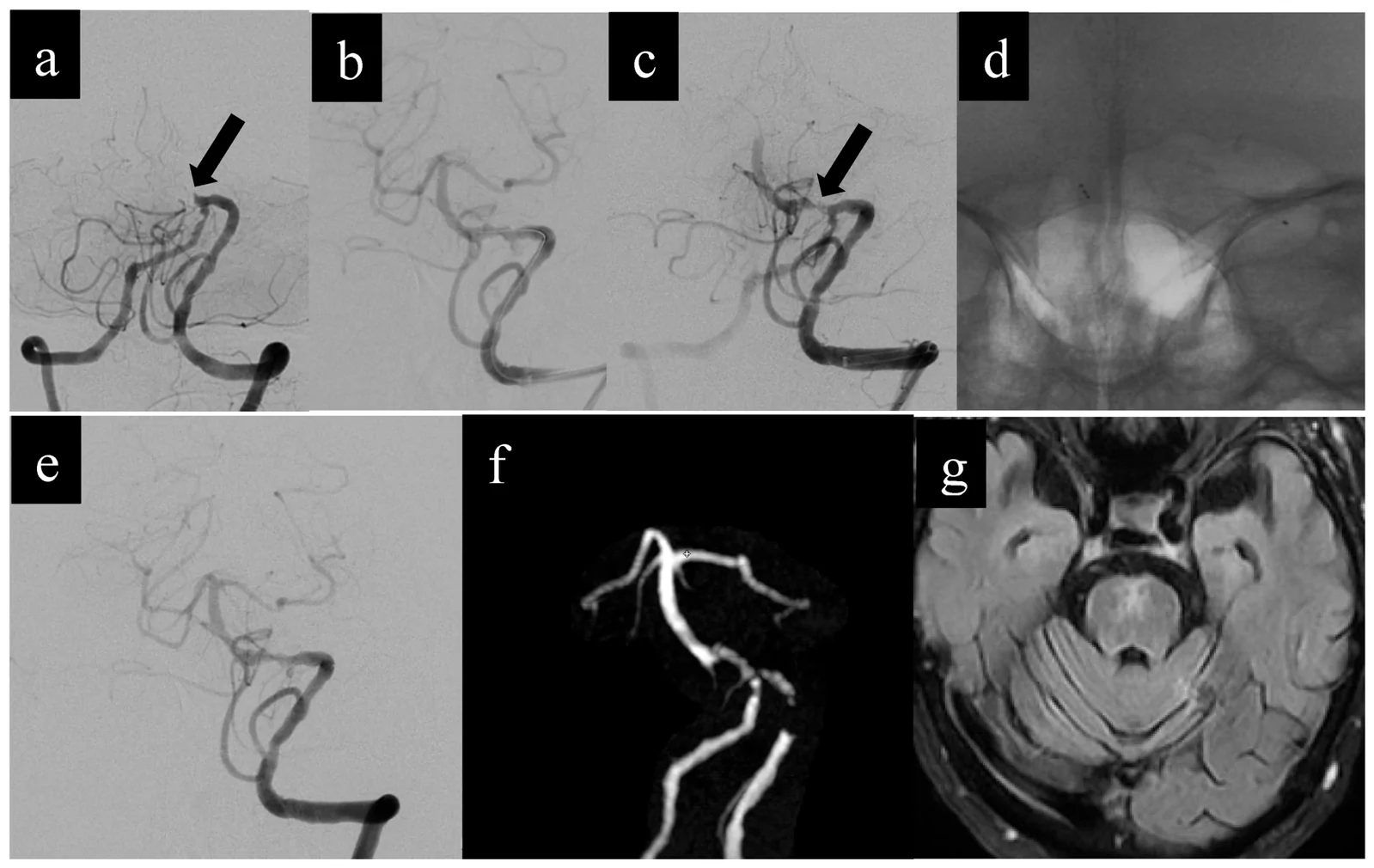
Illustrative case of rescue intracranial stenting for basilar artery occlusion due to intracranial atherosclerotic disease. A 77-year-old man presented with an acute decrease in consciousness for 3 h; the initial NIHSS score was 22. CT angiography demonstrated total occlusion of the basilar artery (BA), and MRI showed no extensive brainstem infarction; the patient was therefore eligible for mechanical thrombectomy. (**a**) Pre-treatment anteroposterior digital subtraction angiogram (DSA) confirmed total occlusion of the proximal BA (arrow). Initial direct contact aspiration and stent-retriever thrombectomy failed to retrieve any clot, suggesting an underlying ICAD lesion as the cause. (**b**) Lateral DSA following balloon angioplasty showed restored BA flow with persistent significant residual stenosis. Intravenous eptifibatide (Hainan Poly Pharm. Co., Ltd., Haikou, China) was administered. (**c**) Lateral DSA 10 min later demonstrated re-thrombosis with diminished antegrade flow, prompting rescue intracranial stenting. (**d**) Anteroposterior fluoroscopic view during deployment of a Solitaire AB stent (4 × 20 mm; Medtronic, Irvine, CA, USA) used as a permanent rescue intracranial stent. (**e**) Lateral DSA 10 min after stent deployment demonstrated complete BA recanalization. (**f**) Three-dimensional time-of-flight MRA at 6-month follow-up showed widely patent flow through the deployed stent. (**g**) Axial fluid-attenuated inversion recovery (FLAIR) MRI at 6 months revealed chronic infarction in the pons and the left cerebellar hemisphere. At the 3-month follow-up, the patient was functionally independent (mRS 0). Abbreviations: BA, basilar artery; DSA, digital subtraction angiography; FLAIR, fluid-attenuated inversion recovery; ICAD, intracranial atherosclerotic disease; MRA, magnetic resonance angiography; MRI, magnetic resonance imaging; mRS, modified Rankin Scale; NIHSS, National Institutes of Health Stroke Scale.

Our cohort reported a 28% prevalence of ICAD, which was associated with poorer outcomes (21% vs. 38% favorable outcomes, *p* = 0.031). This may be explained by the technical challenges associated with ICAD-related occlusions, including higher re-occlusion rates [50] and the need for rescue strategies [50] (Figure 6).

Notably, receipt of intravenous thrombolytic drugs (54% vs. 42%, *p* = 0.200) and the presence of atrial fibrillation (38% vs. 33%, *p* = 0.500) did not show statistically significant associations with outcomes. A previous meta-analysis [51] demonstrated improved functional outcomes with bridging intravenous thrombolysis prior to mechanical thrombectomy compared with direct mechanical thrombectomy, although more recent randomized trials have reported comparable outcomes between the two approaches. A meta-analysis [52] of outcomes in patients with atrial fibrillation following MT found that these patients experienced worse outcomes. In contrast, Akbik et al. [53] reported that patients with atrial fibrillation-associated stroke showed improvement in procedural time and recanalization success without increased intracranial hemorrhage or worse outcomes.

Our findings have several implications for clinical practice, including patient selection, procedural efficiency, anesthetic management, and post-procedural care. Future studies should focus on strategies to improve outcomes in high-risk patients, particularly those involving diabetes management, periprocedural optimization, anesthetic protocols, early extubation, and infection control.

### Strengths and Limitations

The strengths of this study include its prospective design, comprehensive data collection, detailed procedural parameters and thrombus characterization, and multivariate analysis to identify independent predictors.

This study has several limitations. First, the single-center design and modest sample size (*n* = 120; 68 favorable outcomes, 52 unfavorable) constrain the precision of the multivariable estimates. With 52 events in the smaller outcome group, the events-per-variable ratio for the seven-covariate model is approximately 7, below the conventional threshold of 10, and several covariate–outcome cross-tabulations were sparse. We addressed this by specifying covariates a priori rather than by stepwise selection, restricting the model to pre- and intra-procedural variables, and estimating all models by Firth’s penalized-likelihood method with profile likelihood confidence intervals; nevertheless, some intervals remain wide, most notably that for successful recanalization, because only four patients with a favorable outcome failed to recanalize. These estimates should be read as evidence of direction and of the presence of an association rather than as precise effect sizes, and external validation in a larger cohort remains necessary. No adjustment for multiple comparisons was made, so univariate *p*-values should be interpreted descriptively. Second, all imaging assessments (ASPECTS, collateral score, leukoaraiosis grading) were performed by a single neurointerventional radiologist, and histopathological thrombus classification by a single pathologist whose subspecialty training is in thoracic rather than cerebrovascular pathology; no second reader was available, so inter-rater and intra-rater reliability could not be assessed for either domain, and misclassification of thrombus type in particular cannot be excluded. Classification followed a prespecified quantitative threshold (a ≥ 15% difference between erythrocyte and fibrin/platelet content) rather than a subjective impression, which reduces but does not eliminate this concern. The thrombus-composition result should accordingly be regarded as preliminary. Third, 3-month mRS was not observed in 6 patients (5.0%), for whom the 1-month score was carried forward; a complete-case sensitivity analysis gave materially similar results, although baseline ASPECTS reached and baseline NIHSS lost statistical significance under that specification, illustrating the fragility of borderline associations at this sample size. Fourth, all procedures were performed under general anesthesia, which may not reflect practice at all centers and may limit generalizability. Fifth, owing to local facility and personnel constraints, our selection criteria for late-onset stroke were extended up to 12 h, which differs from the DEFUSE 3 trial criteria (6–16 h). Sixth, the observational design precludes definitive causal inferences. Finally, detailed CT perfusion data were available for only 21 patients (17.5%), limiting subgroup analyses related to perfusion-based patient selection.

## 5. Conclusions

This study confirms the efficacy of MT at Siriraj Hospital, with 57% of patients achieving favorable outcomes at 3 months of follow-up, alongside 85% successful recanalization, 7.5% mortality and 3.3% symptomatic intracranial hemorrhage. In a multivariable analysis restricted to variables measurable before or during the procedure and estimated by penalized likelihood, four factors were independently associated with favorable outcome: successful recanalization, absence of diabetes mellitus, lower baseline NIHSS, and red/mixed thrombus composition. Younger age, higher baseline ASPECTS and a minimum intra-procedural diastolic blood pressure ≥ 55 mmHg showed consistent associations that did not reach significance in this cohort and merit examination in larger series. The association with thrombus composition is novel in this population but requires confirmation with immunohistochemical and digital-image methods and independent reading before it could be used prognostically. Beyond these associations, the protocol itself is the more readily transferable finding: outcomes comparable to those of the landmark trials were achieved using non-contrast CT and CT angiography as the default imaging pathway, with perfusion imaging reserved for late-window and uncertain-onset presentations. This is a configuration that does not depend on universal availability of advanced perfusion software or 24 h perfusion staffing, and it may therefore serve as a practical model for stroke centers in other low- and middle-income settings seeking to establish or expand thrombectomy services.

## Figures and Tables

**Table 1:** Summarized table of key clinical and procedural characteristics.

Characteristics, Total *n* = 120	*n* (Percentage)
Demographics	
Gender (female/male)	56 (47%)/64 (53%)
Median age (years)	68 (47–92)
BMI, median (min, max)	24.1 (14.3, 41)
Blood pressure (SBP/DBP), median (min, max)	153 (100, 237)/86 (57, 155)
Clinical presentation	
Median NIHSS at admission, median (min, max)	15 (2, 31)
NIHSS < 16	65 (54%)
Hemiparesis	100 (83%)
Hemiparasthesia	105 (88%)
Aphasia	40 (33%)
Facial palsy	78 (65%)
Altered consciousness	33 (28%)
Comorbidities	
Hypertension	71 (59%)
Diabetes mellitus	35 (29%)
Dyslipidemia	52 (43%)
Atrial fibrillation	43 (36%)
Coronary artery disease	17 (14%)
Previous stroke	20 (17%)
Laboratory results	
Hb (g/dl), median (min, max)	12.75 (8.50, 17.70)
Hct, median (min, max)	39.6 (27.2, 53.6)
WBC (×10^3^/µL), median (min, max)	8.8 (4.2, 23.9)
Neutrophil (×10^3^/µL), median (min, max)	6.0 (0.5, 53.2)
Platelet count (×10^3^/µL), median (min, max)	238 (99, 487)
BUN, median (min, max)	14 (7, 55)
Creatinine, median (min, max)	0.95 (0.40, 9.36)
Triglyceride (mg/dl), median (min, max)	98 (37, 264)
LDL (mg/dl), median (min, max)	94 (31, 295)
Blood sugar (mg/dl), median (min, max)	119 (53, 470)
Stroke characteristics	
Anterior circulation stroke	84 (70%)
Posterior circulation stroke	27 (22.5%)
Tandem occlusion	9 (7.5%)
Imaging findings	
Median ASPECT score, median (min, max)	8 (4, 10)
Collateral score ≥ 4	75/88 (85%)
Hyperdense clot sign	79 (66%)
Leukoaraiosis	52/90 (58%)
Procedure details	
First technique	
Direct thromboaspiration	85 (71%)
Solumbra technique	16 (13.3%)
Stent retrieval technique	12 (10.0%)
Other	7 (5.8%)
First-pass success	50 (42%)
Median puncture-to-recanalization time (min), median (min, max)	23 (3, 123)
Successful recanalization (mTICI ≥ 2B)	102 (85%)
Total procedure time (min), median (min, max)	75 (25, 197)
Outcomes	
Favorable mRS 0–2 at 1 month	63 (53%)
Favorable mRS 0–2 at 3 months	68 (57%)
Median NIHSS at 24 h	7 (0–30)
Symptomatic ICH (sICH)	4 (3.3%)
Sepsis/infection	48 (40%)
No in-hospital complications	43 (36%)
Thrombus characteristics (*n* = 97)	
Thrombus age	
Fresh thrombus (<1 day)	62 (64%)
Lytic (1–5 days)	30 (31%)
Organized (>5 days)	5 (5%)
Thrombus composition	
RBC-rich thrombus	36 (37%)
Mixed thrombus	20 (21%)
White (platelet-rich) thrombus	41 (42%)
Anesthetic factors	
General anesthesia (GA)	120 (100%)
Propofol/Desflurane/sevoflurane	3 (2%)/38 (32%)/79 (66%)
SBP/DBP at puncture (mmHg), median (min, max)	121 (75, 183)/71 (42, 108)
Maximum SBP/DBP during procedure (mmHg), median (min, max)	162 (115, 230)/93 (55, 146)
Minimum SBP/DBP during procedure (mmHg), median (min, max)	93 (70, 133)/56 (30, 83)
Extubation within 24 h	83 (69%)

Data are presented as *n* (%) for categorical variables and median (minimum, maximum) for continuous variables, unless otherwise stated. Unless indicated, percentages are calculated using the full cohort (*N* = 120) as the denominator. Anterior circulation, posterior circulation and tandem occlusion are mutually exclusive categories. Denominators differ from 120 for variables that were not assessable in all patients: baseline ASPECTS was scored in 107 patients, collateral score in 88, leukoaraiosis in 90, and thrombus histopathology in 97 (thrombus was not retrievable in 23 patients); percentages for these variables use the number assessed as the denominator. Within the thrombus characteristics, the age and composition subcategories are separate classifications, and each sums to 100% of the 97 specimens examined. Anesthetic maintenance was with desflurane in 38 patients (32%) and sevoflurane in 79 (66%); the remaining 3 patients (2%) received an alternative maintenance regimen. Complication categories are not mutually exclusive, as individual patients could experience more than one event. Abbreviations: ASPECTS, Alberta Stroke Program Early CT Score; BMI, body mass index; BUN, blood urea nitrogen; DBP, diastolic blood pressure; GA, general anesthesia; Hb, hemoglobin; Hct, hematocrit; LDL, low-density lipoprotein; mRS, modified Rankin Scale; mTICI, modified Thrombolysis in Cerebral Infarction; NIHSS, National Institutes of Health Stroke Scale; RBC, red blood cell; SBP, systolic blood pressure; sICH, symptomatic intracranial hemorrhage; WBC, white blood cell.

**Table 2:** Summarized table of factors associated with favorable outcomes in the univariate analysis.

Factors	Unfavorable Outcome (*n* = 52)	Favorable Outcome (*n* = 68)	*p*-Value
Male gender, *n* (%)	22 (42%)	42 (62%)	0.034
Age, median (min, max)	71 (48, 91)	65 (47, 92)	0.008
Age ≥ 65 years, *n* (%)	39 (75%)	35 (51%)	0.009
Pulse pressure, median (min, max)	73 (15, 155)	62 (27, 129)	0.021
Initial wide pulse pressure, *n* (%)	16 (31%)	10 (15%)	0.034
DM, *n* (%)	24 (46%)	11 (16%)	<0.001
Atrial fibrillation, *n* (%)	17 (33%)	26 (38%)	0.5
NIHSS, median (min, max)	17 (6, 31)	13 (2, 31)	0.005
NIHSS < 16, *n* (%)	22 (42%)	43 (63%)	0.023
Alteration of consciousness, *n* (%)	21 (40%)	12 (18%)	0.006
Facial palsy, *n* (%)	29 (56%)	49 (72%)	0.064
Dysarthria, *n* (%)	19 (37%)	39 (57%)	0.024
GCS, median (min, max)	11.00 (3.00, 15.00)	15.00 (8.00, 15.00)	<0.001
Blood sugar ≥ 200 mg/dl, *n* (%)	10 (19%)	2 (2.9%)	0.003
Blood sugar (mg/dl), median (min, max)	137 (53, 470)	115 (65, 242)	<0.001
BUN, median (min, max)	15 (7, 42)	13 (7, 55)	0.064
Platelet count (×10^3^/µL), median (min, max)	246 (99, 487)	234 (130, 416)	0.070
Leukoaraiosis on imaging, *n* (%)	31/42 (74%)	21/48 (44%)	0.004
First-pass success, *n* (%)	16 (31%)	34 (50%)	0.041
Successful recanalization (mTiCI ≥ 2B), *n* (%)	38 (73%)	64 (94%)	0.001
No abnormality on CBCT, *n* (%)	36 (69%)	59 (87%)	0.019
ICAD, *n* (%)	20 (38%)	14 (21%)	0.031
rtPA received, *n* (%)	22 (42%)	37 (54%)	0.2
Onset to door (minutes), median (min, max)	157 (16, 715)	148 (5, 845)	0.9
Puncture to recanalization (≥2B) (minutes), median (min, max)	30 (3, 88)	21 (4, 123)	0.032
Total procedure time (arrival to closure time), median (min, max)	81 (25, 197)	69 (25, 165)	0.020
Immediate postoperative NIHSS, median (min, max)	16 (3, 29)	7 (1, 22)	<0.001
24 h NIHSS, median (min, max)	14 (1, 30)	3 (0, 18)	<0.001
Postoperative NIHSS at 24 h < 16, *n* (%)	31 (60%)	67 (99%)	<0.001
72 h NIHSS, median (min, max)	13 (0, 30)	2 (0, 11)	<0.001
NIHSS before d/c, median (min, max)	12 (0, 31)	1 (0, 9)	<0.001
Visual ASPECT score in 24 h CT, median (min, max)	6 (0, 9)	8 (3, 10)	<0.001
No significant abnormality on 24 h CT brain, *n* (%)	31 (60%)	66 (97%)	<0.001
sICH, *n* (%)	4 (7.7%)	0 (0%)	0.033
Postoperative sepsis/infection, *n* (%)	30 (58%)	18 (26%)	<0.001
Recurrent stroke, *n* (%)	6 (12%)	0 (0%)	0.006
No postoperative complication, *n* (%)	6 (12%)	37 (54%)	<0.001
Thrombus composition: RBCs %, median (min, max)	40 (5, 95)	50 (0, 90)	0.015
Thrombus composition: fibrin and platelets %, median (min, max)	48 (5, 85)	30 (0, 70)	0.002
White thrombus, *n* (%)	23/38 (61%)	18/59 (31%)	0.003
SBP at puncture (mmHg), median (min, max)	119 (75, 170)	125 (88, 183)	0.067
DBP at puncture (mmHg), median (min, max)	67 (42, 96)	75 (44, 108)	<0.001
Minimum SBP during procedure (mmHg), median (min, max)	92 (70, 133)	93 (74, 127)	0.4
Minimum DBP during procedure (mmHg), median (min, max)	55 (30, 82)	57 (38, 83)	0.018
Post-reperfusion DBP, median (min, max)	65 (43, 96)	70 (45, 107)	0.046
Induction with succinyl choline, *n* (%)	25 (48%)	49 (72%)	0.007
Extubation within 24 h, *n* (%)	23 (44%)	60 (88%)	<0.001
Extubation within 72 h, *n* (%)	36 (69%)	67 (99%)	<0.001

Data are presented as *n* (%) for categorical variables and median (minimum, maximum) for continuous variables. A favorable outcome was defined as a modified Rankin Scale score of 0–2 at 3 months. Percentages are calculated within each outcome group (unfavorable, *n* = 52; favorable, *n* = 68) unless a different denominator is indicated. Leukoaraiosis was assessable in 90 patients (42 in the unfavorable and 48 in the favorable group) and thrombus histopathology was available in 97 (38 and 59, respectively); percentages for these two variables use the number assessed as the denominator. Continuous variables were compared using the Wilcoxon rank-sum test and categorical variables using Pearson’s chi-squared test or Fisher’s exact test, as appropriate. No adjustment was made for multiple comparisons, and *p*-values are therefore descriptive rather than confirmatory. Variables measured after the procedure—the 24 h, 72 h and pre-discharge NIHSS, findings on 24 h CT, and the timing of extubation—are reported here as measures of early neurological response and were not entered as predictors in the multivariable model. Abbreviations: CBCT, cone-beam computed tomography; d/c, discharge; DBP, diastolic blood pressure; GCS, Glasgow Coma Scale; ICAD, intracranial atherosclerotic disease; mTICI, modified Thrombolysis in Cerebral Infarction; NIHSS, National Institutes of Health Stroke Scale; rtPA, recombinant tissue plasminogen activator; SBP, systolic blood pressure; sICH, symptomatic intracranial hemorrhage.

**Table 3:** Firth penalized-likelihood multivariable analysis of pre- and intra-procedural predictors of favorable outcome (mRS 0–2 at 3 months) in the thrombus-pathology subgroup (*n* = 88; 54 favorable, 34 unfavorable). Odds ratios are shown with profile penalized-likelihood 95% confidence intervals and penalized likelihood-ratio *p*-values. All variance inflation factors were <1.3. ^a^ Male sex and induction with succinylcholine are reported from the prespecified sensitivity model and were not part of the primary model.

Factors	Adjusted OR	*p*-Value	Confidence Interval
Successful recanalization (mTICI ≥ 2b)	19.51	0.004	2.47–274.05
Absence of diabetes mellitus	6.22	0.008	1.58–32.87
Baseline NIHSS (per 1-point increase)	0.87	0.005	0.76–0.96
Red/mixed thrombus composition	3.06	0.049	1.01–9.96
Minimum diastolic BP during procedure ≥ 55 mmHg	3.28	0.058	0.96–13.07
Age (per 1-year increase)	0.95	0.054	0.89–1.00
Baseline ASPECTS (per 1-point increase)	1.29	0.166	0.90–1.88
Male sex ^a^	1.22	0.748	0.36–4.08
Induction with succinylcholine ^a^	2.24	0.184	0.68–7.91

## Data Availability

The data presented in this study are available on request from the corresponding author, due to privacy restrictions.
